# Combined Medication of Antiretroviral Drugs Tenofovir Disoproxil Fumarate, Emtricitabine, and Raltegravir Reduces Neural Progenitor Cell Proliferation In Vivo and In Vitro

**DOI:** 10.1007/s11481-017-9755-4

**Published:** 2017-07-22

**Authors:** Peipei Xu, Yingchun Wang, Zhao Qin, Lisha Qiu, Min Zhang, Yunlong Huang, Jialin C. Zheng

**Affiliations:** 1 Center for Translational Neurodegeneration and Regenerative Therapy, Shanghai Tenth People’s Hospital Affiliated with Tongji University School of Medicine, Shanghai, 200072 China; 2 Department of Neurology, Shanghai Tongji Hospital, Tongji University School of Medicine, Shanghai, 200065 China; 3 Departments of Pharmacology and Experimental Neuroscience, University of Nebraska Medical Center, Omaha, NE 68198-5930 USA; 4 Department of Pathology and Microbiology, University of Nebraska Medical Center, Omaha, NE 68198-5930 USA

**Keywords:** TDF, FTC, RAL, Neural progenitor cells

## Abstract

The application of combination antiretroviral therapy has greatly reduced the death rate from AIDS. However, up to 50% of patients on combination antiretroviral therapy develop HIV-associated neurocognitive disorders (HAND), which is associated with residual neuroinflammation and oxidative injury in the brain. Neural stem cells (NSCs) and progenitors play a vital role in repairing neuronal injuries. Therefore, we hypothesize that combination antiretroviral therapy may adversely affect NSCs/progenitors, contributing to the increasing prevalence of HAND. Here, we show that combined medication of three antiretroviral drugs tenofovir disoproxil fumarate (TDF), emtricitabine (FTC), and raltegravir (RAL) affects NSC homeostasis and progenitor proliferation in the mouse dentate gyrus (DG). Our results also show that TDF/FTC/RAL treatment prohibits proliferation and induces apoptosis of cultured mouse neural progenitor cells (NPCs), resulting in a reduction in the viability of NPCs. Moreover, we find that TDF, among the three drugs used in this combination antiretroviral treatment, accounts for most of the effects on neural progenitors. Together, our results offer a mechanistic explanation for the prevalence of HAND in AIDS patients treated with combination antiretroviral therapy.

**Electronic supplementary material:**

The online version of this article (doi:10.1007/s11481-017-9755-4) contains supplementary material, which is available to authorized users.

## Introduction

The international treatment guidelines for AIDS recommend combined medication of three antiretroviral drugs (a drug “cocktail”) to achieve sufficient suppression of HIV-1 RNA replication: two nucleoside reverse transcriptase inhibitors (NRTIs), usually tenofovir disoproxil fumarate (TDF) and emtricitabine (FTC), plus either an integrase strand transfer inhibitor (INSTI), a non-nucleoside reverse transcriptase inhibitor, or a boosted protease inhibitor (Labarga 2015). NRTIs bind to the HIV-1 reverse transcriptase and inhibit proviral DNA synthesis. INSTIs are potent inhibitors of the HIV-1 integrase. They block the HIV-1 genetic material from attaching to the host cell’s DNA (Gunthard et al. 2014). A commonly used INSTI in anti-HIV “cocktail” treatment is raltegravir (RAL). The application of combination antiretroviral therapy has effectively reduced the death rate from AIDS. If treated, patients infected with HIV are now expected to have nearly normal life expectancy (Bhatti et al. 2016; von Braun et al. 2014).

HIV-1 can enter the central nervous system (CNS) during early stages of infection (Nath and Sacktor 2006). CNS HIV infection frequently results in a neurological condition marked by a set of cognitive, motor, and behavioral symptoms known as HIV-associated neurocognitive disorders (HAND) (Antinori et al. 2007). HAND is associated with chronic inflammation and oxidative injury in the brain (Del Guerra et al. 2013; Fischer-Smith and Rappaport 2005; Rao et al. 2014). Unfortunately, combination antiretroviral therapy does not seem to help in controlling the progression of HAND: patients still develop cognitive impairment even though their plasma HIV load is under control (McArthur et al. 2005; Sacktor 2002). As a result, the prevalence of HAND is increasing, affecting up to 50% of patients on combination antiretroviral therapy (Heaton et al. 2011; Robertson et al. 2007).

The underlying neuropathogenesis of HAND remain elusive. Neural stem cells (NSCs) and progenitors are known to have the ability to produce neuroblasts that migrate to areas of brain injuries and replace lost neurons (Aboody et al. 2000; Imitola et al. 2004; Imitola et al. 2003; Park et al. 2002a; Park et al. 2002b; Saha et al. 2013; Snyder et al. 1997). Recent studies showed that administration of each of two antiretroviral drugs, zidovudine and efavirenz (a HIV-1 non-nucleoside reverse transcriptase inhibitor) leads to severe perturbations in both the proliferative and neurogenic capacities of NSCs/progenitors (Demir and Laywell 2015; Jin et al. 2016). Therefore, we hypothesize that anti-HIV-1 “cocktail” treatment may also adversely affect NSCs/progenitors, contributing to the increasing prevalence of HAND. In this paper, we investigated this possibility. We found that combined medication of TDF, FTC, and RAL affects NSC homeostasis and progenitor proliferation in the mouse dentate gyrus (DG). To further understand the mechanism, we tested the effect of TDF/FTC/RAL treatment on cultured mouse neural progenitor cells (NPCs) and found that combined TDF/FTC/RAL medication inhibits proliferation and induces apoptosis of NPCs in a dose- and time-dependent manner. We also showed that TDF, one of the two NRTIs used in the three drug “cocktail”, accounts for most of the effects of combination antiretroviral therapy on NPCs.

## Materials and Methods

### Mice

The C57BL/6 mice were purchased from the Model Animal Research Center of Nanjing University. All mice were housed in the Comparative Medicine facilities of Tongji University School of Medicine. All procedures were conducted according to protocols approved by the Institutional Animal Care and Use Committee of Tongji University School of Medicine.

### Drug Treatment

For in vivo studies, 10-week-old C57BL/6 mice were randomly assigned to two groups (*n* = 6 for each group). One group received TDF/FTC/RAL combined medication (104/120/28 mg/kg, TDF and RAL were dissolved in DMSO, FTC in 0.9% NaCl; Shengda Pharmaceutical Co., Limited, China) while the other received vehicle control (DMSO and 0.9% NaCl) via daily intraperitoneal (i.p.) injections for 60 days. The dose used in this study is within the range of drug concentrations used in other mouse studies (Denton et al. 2012) and mice were weighed daily to adjust drug intake.

For in vitro studies, mouse NPCs were treated with antiretroviral drugs (dissolved in DMSO; MedChem Express) in combination or individually at various concentrations. 1×: 1 μg/ml for TDF, 2 μg/ml for FTC, and 0.1 μg/ml for RAL. 0.1×, 0.3×, 0.5×, 3×, 5×, and 10× were calculated based on 1× concentrations. Control group was treated with DMSO (0.55 mg/ml; Sigma-Aldrich).

### Quantification of BrdU-Positive Cells in the Mouse DG

For long-term BrdU labeling experiments, BrdU (Invitrogen) was injected along with the drugs at 5 μl/g for 5 consecutive days from the first day of drug treatment. For short-term BrdU labeling experiments, BrdU was injected at 10 μl/g 2 h before euthanization on the day following the last drug treatment. Mice were anesthetized with 4% chloral hydrate and transcardially perfused with cold PBS followed by 4% paraformaldehyde (PFA). Brain tissues were removed, fixed in 4% PFA at 4 °C for 48 h, and cryoprotected in 30% sucrose for 48 h before sectioning. Fixed, cryoprotected brains were frozen and sectioned in the horizontal plane at 30 μm intervals using a Cryostat (Leica). In order to go through the entire hippocampus, 10 sections were collected for each mouse. Sections were placed on glass slides and air dried. For BrdU immunohistochemistry, sections were incubated with 0.4% pepsase for 10 min and denatured with 2 M hydrochloric acid for 30 min at 37 °C. Then sections were permeabilized with 0.1% Triton X-100 in PBS for 20 min, blocked with 5% goat serum for 1 h, and incubated with mouse anti-BrdU antibody (BD Biosciences) at 1:500 dilution overnight at 4 °C. After washing, sections were incubated with secondary antibody (Alexa Fluor 488-conjugated goat anti-mouse IgG, 1:500; Invitrogen) for 1 h. Nuclear DNA was labeled with 4′, 6-diamidino-2-phenylindole (DAPI; Sigma-Aldrich) for 10 min. Sections were mounted in mounting medium (Sigma-Aldrich) and fluorescence was examined by a Zeiss META 710 confocal microscope. Images were analyzed using Image-ProPlus, version 7.0 and the number of BrdU-positive cells in the entire dentate gyrus was determined for each mouse.

### Mouse NPC Culture and Immunohistochemistry

The forebrain of each mouse embryo at E13.5 was dissected and mechanically dissociated. Cells from each forebrain were seeded into a 100 mm Petri dish at a density of 2 × 10^5^ cells/ml in 10 ml of mouse NeuroCult NSC Proliferation Medium (Stem Cell Technologies) supplemented with epidermal growth factor (20 ng/ml; Gibco) and basic fibroblast growth factor (10 ng/ml; Gibco) for selective neurosphere growth. Neurospheres were passaged when they reached 100–150 μm in diameter. For immunochemistry, mouse NPCs were grown in 35 mm glass-bottom dishes (MatTek) at a density of 8 × 10^4^ cells/ml for 24 h, fixed using 4% PFA, and permeabilized with 0.4% Triton-X in PBS. Subsequently, they were incubated overnight at 4 °C with primary antibodies including mouse anti-Ki67 (1:200; Cell Signaling) and chicken anti-Nestin (1:500; Novus) for the identification of proliferating NPCs. This was followed by incubation with secondary antibodies: goat anti-mouse IgG (conjugated with Alexa Flour 488; Invitrogen) and goat anti-chicken IgG (conjugated with Alexa Fluor 568; Invitrogen). Nuclei were counter-stained with DAPI. Immunostaining was examined by a Zeiss META 710 confocal microscope and images were imported into Image-ProPlus, version 7.0 for qualification.

### Western Blotting

Mouse NPCs were lysed by M-PER Protein Extraction Buffer (Pierce). Total protein concentration was determined using the Bicinchoninic Acid (BCA) Protein Assay Kit (Pierce). Analytical SDS-polyacrylamide gel electrophoresis (SDS-PAGE) was performed using 10% and 15% gels. Proteins were then transferred onto an Immuno-Blot polyvinylidene fluoride membrane (Bio-Rad). After blocked in 5% fat-free milk for 1 h, the membrane was incubated with primary antibodies for Caspase-3 (1:1000; Cell Signaling Technologies), poly ADP-ribose polymerase (PARP, 1:1000; Cell Signaling Technologies), and Actin (1:5000; Sigma-Aldrich) overnight at 4 °C followed by horseradish peroxidase-conjugated secondary antibodies for 1 h at room temperature. Protein signals were detected using a chemiluminescent substrate solution. The density of each band was determined by Image Lab software and analyzed using Image J program.

### Cell Viability Assay

Cell viability was determined using the Cell Counting Kit-8 (CCK-8; YEASEN). Mouse NPCs were seeded on 96-well plates with a density of 1 × 10^4^ cells per well. After overnight incubation, cells were treated with either DMSO (0.55 mg/ml, negative control), cytosine β-D-arabinofuranoside (Ara-C, 7 μg/ml, positive control; Sigma), or various concentrations of antiretroviral drugs (0.1×, 0.3×, 0.5×, and 1×). Half of the medium liquid was renewed every three days. On day 2, 4, 6, and 8, 10 μl CCK-8 solution was added into each well of cell culture and the plates were incubated for another 2 h at 37 °C. The optical density was then measured at an absorbance of 450 nm using a microplate reader (DynaMax Biotech Co., Ltd.). Culture medium without cells served as a blank control. Cell viability was calculated using the following equation: cell viability = (OD _drug treated group_ - OD _blank_) / (OD _DMSO treated group_ - OD _blank_). Experiments were performed in triplicates and repeated at least three times independently.

### Statistical Analysis

Statistical analysis was performed on all quantitative assays. Data were presented as mean ± SEM. The appropriate one-way ANOVA was used to determine statistical significance with *p* < 0.05 considered significant (GraphPad Prism). All experiments were performed with at least three donors to account for any donor specific differences. Assays were performed at least three times in triplicates or quadruplicates.

## Results

### TDF/FTC/RAL Combined Medication Affects NSC Homeostasis and Progenitor Proliferation in the Mouse DG*.*

**Fig. 1:**
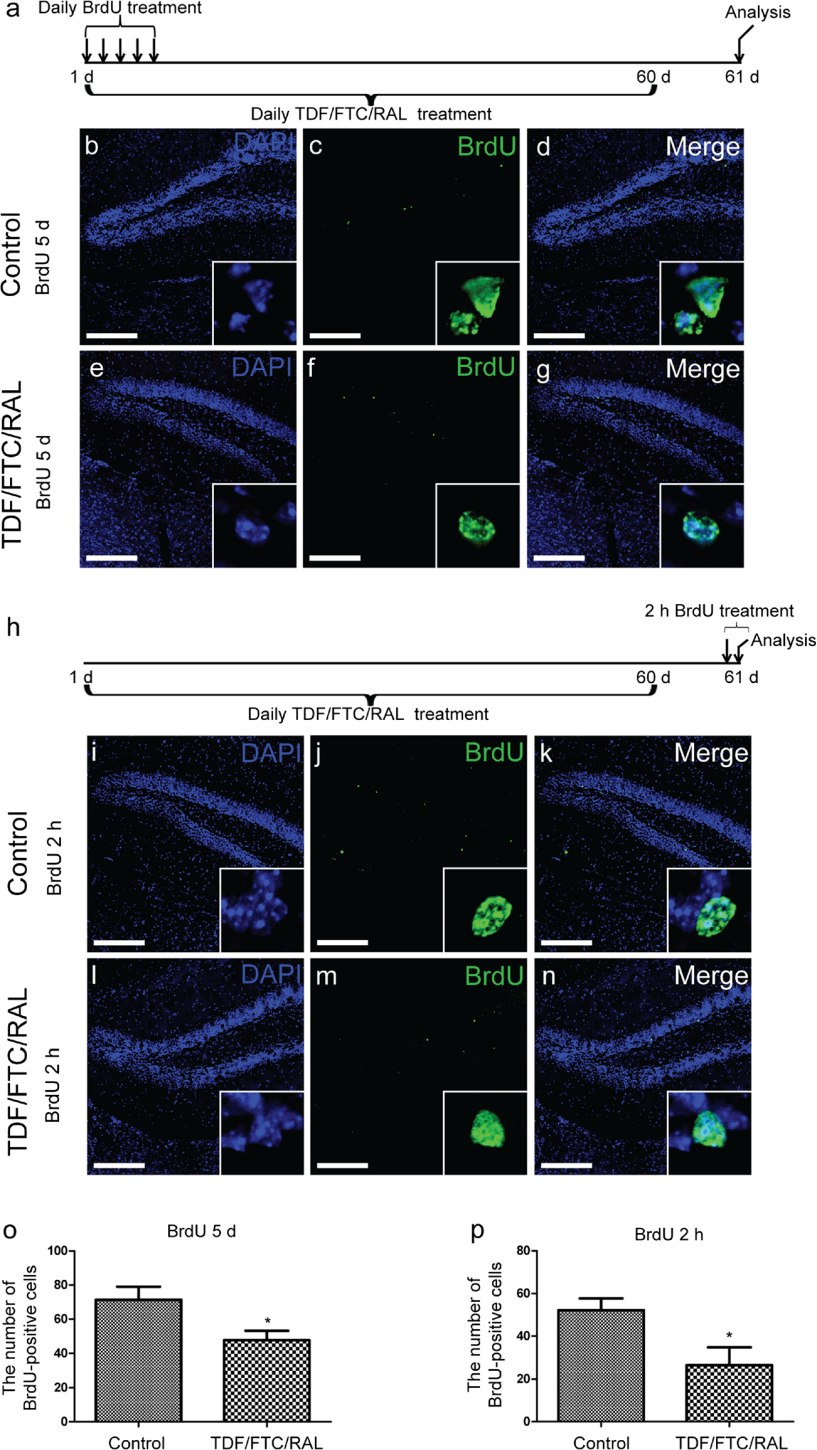
TDF/FTC/RAL combined medication affects NSC homeostasis and progenitor proliferation in the mouse DG. 10-week-old C57BL/6 mice were injected daily with either TDF/FTC/RAL (104/120/28 mg/kg) or vehicle control for 60 days. **a** Experimental design for long-term BrdU labeling experiments. BrdU was injected along with the drugs at 5 μl/g for 5 consecutive days from the first day of drug treatment. **b-g** Representative confocal microscope images of coronal sections through the mouse hippocampal DG immunolabeled for DAPI (*blue*) and BrdU (*green*). Bottom right corner of each image shows the BrdU-positive cells in the molecular layer of the DG. **h** Experimental design for short-term BrdU labeling experiments. BrdU was injected at 10 μl/g 2 h before euthanization on the day following the last drug treatment. **i-n** Representative confocal microscope images of coronal sections through the mouse hippocampal DG immunolabeled for DAPI (*blue*) and BrdU (*green*). Bottom right corner of each image shows the BrdU-positive cells in the molecular layer of the DG. Quantification of total BrdU-positive cells was shown in **o** and **p**. * denotes *p* < 0.05. Scale bar: 200 μm. *n* = 6 for each group

To examine the effect of TDF/FTC/RAL combined medication on the NSC pool in vivo, we performed long-term BrdU labeling experiments. 10-week-old C57BL/6 mice were injected daily with either TDF/FTC/RAL or vehicle control for 60 days and BrdU was administered along with the drugs for the first 5 days. The mice were sacrificed the day after the last drug injection and the number of BrdU-retaining cells in the DG was quantified (Fig. 1a). These BrdU-retaining, slow-cycling cells are thought to be the relatively quiescent NSCs (Bondolfi et al. 2004). We found that TDF/FTC/RAL treatment caused a significant reduction in the number of labeling-retaining NSCs (Fig. 1b-g and o), indicating that TDF/FTC/RAL combined medication affects NSC homeostasis in vivo.

To determine if TDF/FTC/RAL medication affects proliferation of neural progenitors in the DG, we performed short-term BrdU labeling experiments. 10-week-old C57BL/6 mice were treated with either TDF/FTC/RAL or vehicle control for 60 days and BrdU was injected into the mice 2 h before euthanization on the day following the last drug treatment (Fig. 1h). We found that TDF/FTC/RAL treated mice had considerably fewer BrdU-postive cells in the DG than the control group (Fig. 1i-n and p). This result suggests that TDF/FTC/RAL negatively regulates neural progenitor proliferation in vivo.

### TDF/FTC/RAL Combined Medication Reduces Viability and Proliferation of Mouse NPCs In Vitro.

**Fig. 2:**
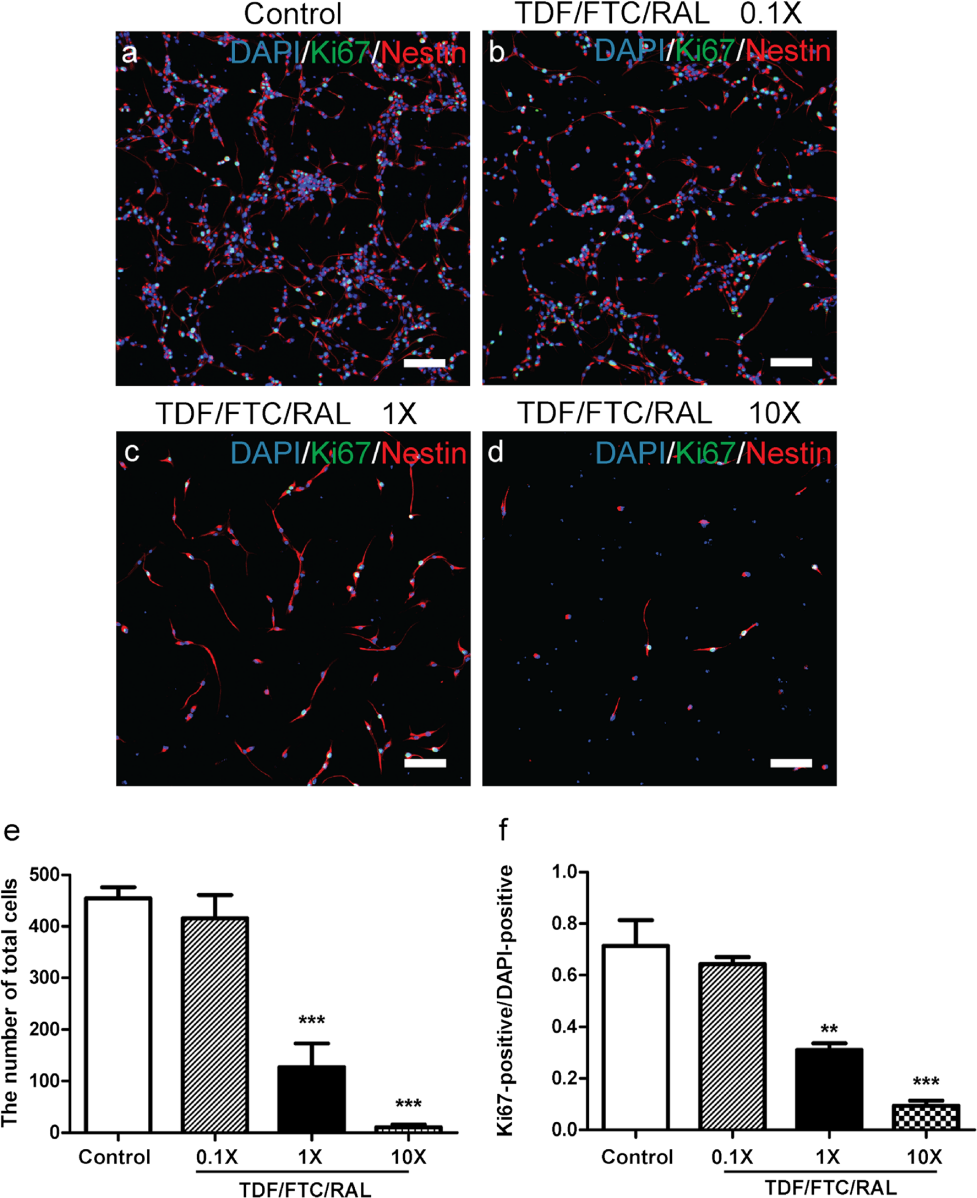
TDF/FTC/RAL combined medication reduces viability and proliferation of mouse NPCs in vitro. Mouse NPCs were treated with either DMSO **a** or doses of TDF/FTC/RAL **b-d**. After 48 h, cells were fixed and stained with DAPI (*blue*), anti-Ki67 (*green*), and anti-Nestin (*red*) antibodies. Quantification of total cell number was shown in **e**. Quantification of the ratio between Ki67-positive and DAPI-positive cells was shown in **f**. ** denotes *p* < 0.01, *** denotes *p* < 0.001. Scale bar: 100 μm

To investigate the cellular mechanisms by which TDF/FTC/RAL affects neural progenitors, we treated cultured mouse NPCs with TDF/FTC/RAL at low, moderate, and high concentrations (0.1×, 1×, and 10×, respectively) for 48 h. 1× represents the peak plasma concentration for each drug in clinical practices (Calza et al. 2015; Gomes et al. 2008). Control group was treated with DMSO. We co-stained the cells with DAPI, a nuclear DNA marker, Ki67, a cell proliferation marker, and Nestin, a NPC marker. We found that the number of NPCs was significantly reduced by moderate to high doses of TDF/FTC/RAL treatment (Fig. 2e). This result suggests that TDF/FTC/RAL combined medication reduces viability of cultured mouse NPCs. In addition, we found that the ratio between Ki67-positive and DAPI-positive cells was similarly affected by TDF/FTC/RAL treatment (Fig. 2f), indicating that TDF/FTC/RAL combined medication also reduces mouse NPC proliferation in vitro. Together, these results are consistent with what we found in our previous in vivo studies.

### TDF/FTC/RAL Combined Medication Induces Mouse NPC Apoptosis In Vitro.

**Fig. 3:**
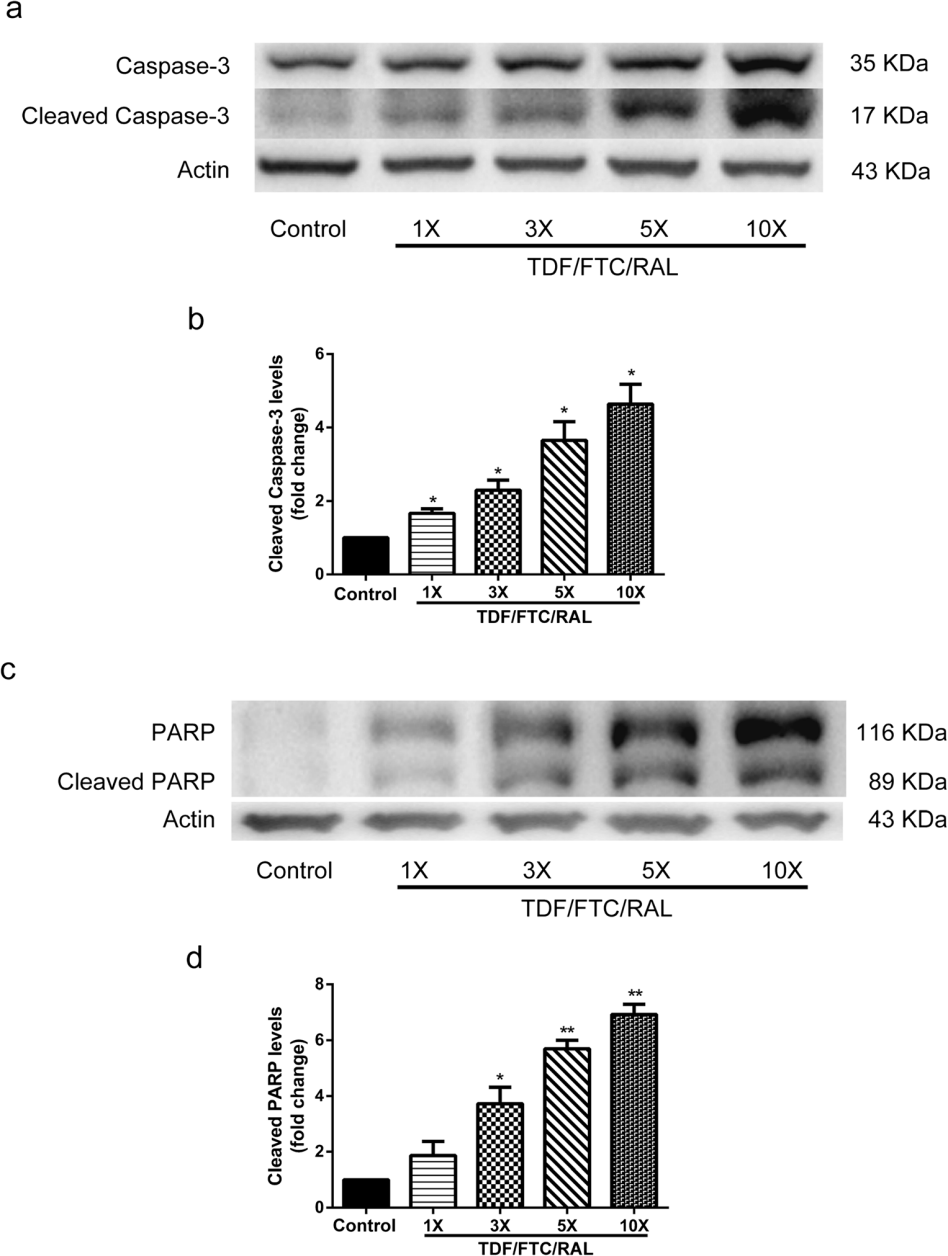
TDF/FTC/RAL combined medication induces mouse NPC apoptosis in vitro. Mouse NPCs were treated with either DMSO or TDF/FTC/RAL for 8 h. **a** Cleaved Caspase-3 levels were determined by Western blotting. **b** Levels of cleaved Caspase-3 were normalized to the levels of Actin and shown as fold changes relative to the control. **c** Cleaved PARP levels were determined by Western blotting. **d** Levels of cleaved PARP were normalized to the levels of Actin and shown as fold changes relative to the control. * denotes *p* < 0.05, ** denotes *p* < 0.01

To determine if apoptosis also accounts for the loss of NPCs in the above analysis, we measured cell apoptotic markers, cleaved Caspase-3 and cleaved poly ADP-ribose polymerase (PARP). Caspase-3 has been reported in several studies as the executioner of apoptosis (Slee et al. 2001; Walsh et al. 2008). PARP is cleaved by the cleaved Caspase-3, hence restricting DNA repair (Wurzer et al. 2003). We performed Western blotting to identify cleavage products of Caspase-3 and PARP in cultured mouse NPCs treated with either DMSO or TDF/FTC/RAL from moderate to high doses (1×, 3×, 5×, and 10×) for 8 h. We found that the levels of cleaved Caspase-3 and cleaved PARP were up-regulated in groups treated with TDF/FTC/RAL, and the magnitude of the up-regulation was proportional to the drug concentration (Fig. 3). Therefore, these data suggest that combined medication of TDF, FTC, and RAL induces mouse NPC apoptosis in vitro.

### TDF/FTC/RAL Combined Medication Affects Mouse NPCs in a Dose- and Time-Dependent Manner.

**Fig. 4:**
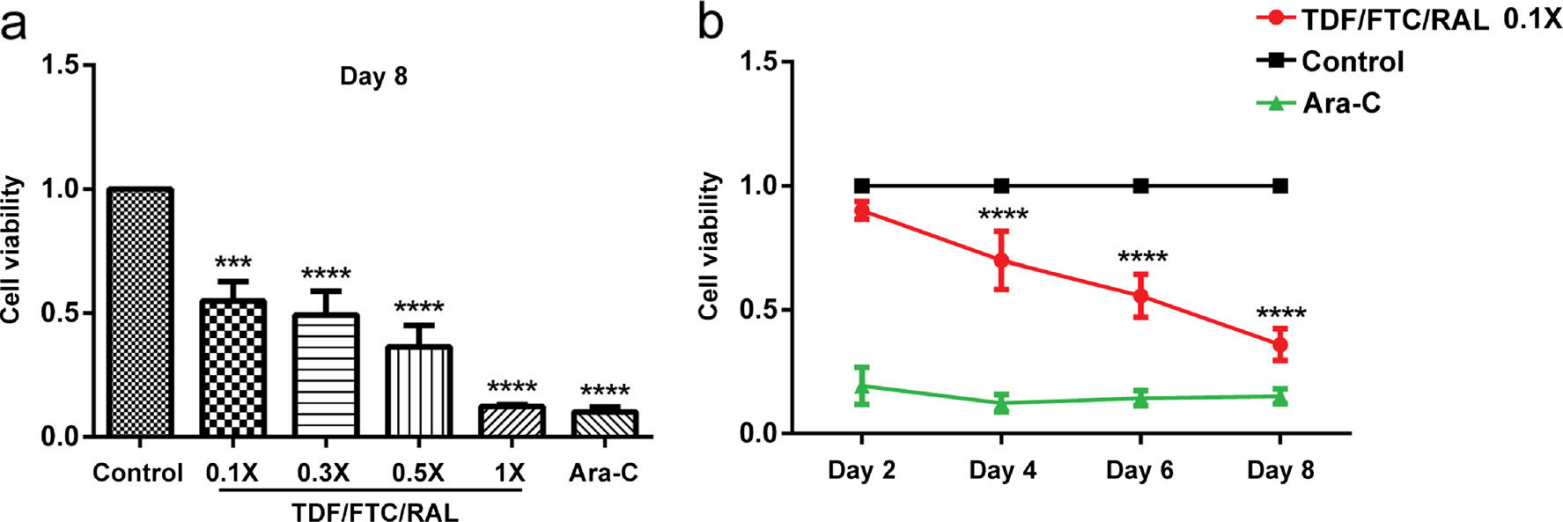
TDF/FTC/RAL combined medication affects mouse NPCs in a dose- and time-dependent manner. Viability of cultured mouse NPCs was determined using the CCK-8. **a** Mouse NPCs were treated with increasing concentrations of TDF/FTC/RAL (0.1×, 0.3×, 0.5×, and 1×), and cell viability was measured on day 8. **b** Mouse NPCs were treated with 0.1× TDF/FTC/RAL and cell viability was determined on day 2, 4, 6, and 8. Control groups were treated with DMSO. Ara-C (7 μg/ml) served as a positive control. *** denotes *p* < 0.001, **** denotes *p* < 0.0001 compared with control

Given that AIDS patients are treated with antiretroviral drugs for a long time, we then wondered if TDF/FTC/RAL could affect the viability of NPCs at lower concentrations by increasing the time of exposure. We treated cultured mouse NPCs with doses of TDF/FTC/RAL equal or less than 1× (0.1×, 0.3×, 0.5×, and 1×), and performed cell viability assays on day 2, 4, 6, and 8 using the CCK-8. Ara-C, a DNA synthesis inhibitor, was used as a positive control. We found that TDF/FTC/RAL treatment reduced the viability of cultured mouse NPCs, consistent with our previous analyses. Moreover, our results showed that the magnitude of the reduction was proportional to both the TDF/FTC/RAL concentration (Fig. 4a) and the time of exposure to the drugs (Fig. 4b), indicating that TDF/FTC/RAL combined medication affects mouse NPCs in a dose- and time-dependent manner.

### TDF Alone Reduces Viability and Proliferation of Mouse NPCs.

**Fig. 5:**
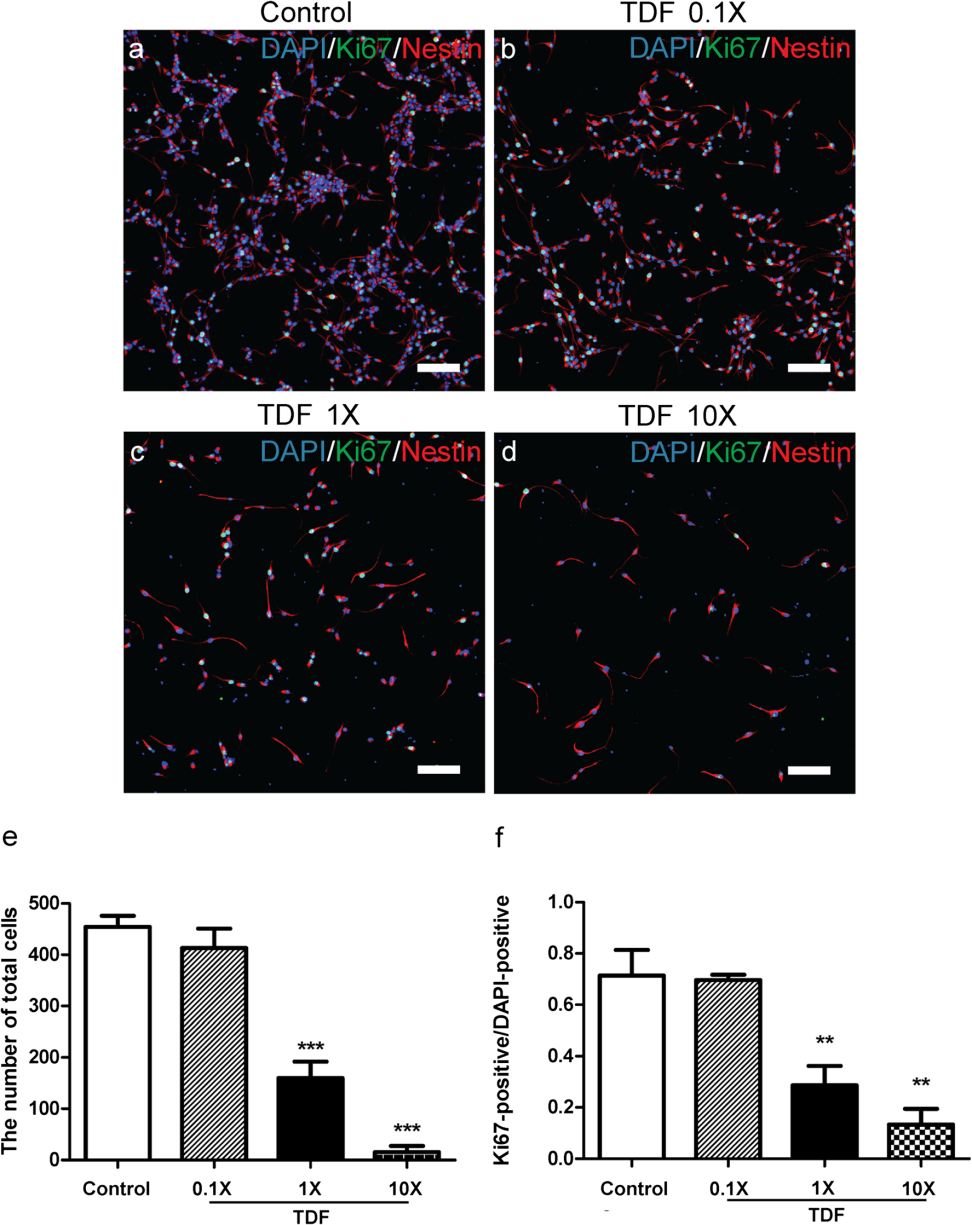
TDF alone reduces viability and proliferation of mouse NPCs. Mouse NPCs were treated with DMSO **a** or TDF **b-d**. After 48 h, cells were fixed and stained with DAPI (*blue*), anti-Ki67 (*green*), and anti-Nestin (*red*) antibodies. Quantification of total cell number was shown in **e**. Quantification of the ratio between Ki67-positive and DAPI-positive cells was shown in **f**. ** denotes *p* < 0.01, *** denotes *p* < 0.001. Scale bar: 100 μm

To examine if any of the three drugs alone could affect neural progenitors, we first treated cultured mouse NPCs with TDF, FTC, and RAL individually at low, moderate, and high concentrations (0.1×, 1×, and 10×) for 48 h and co-stained the cells with DAPI, Ki67, and Nestin. We found that TDF treatment at 1× and 10× concentrations significantly reduced both the number of DAPI-positive cells and the ratio between Ki67-positive and DAPI-positive cells (Fig. 5f). In contrast, FTC and RAL had no effect even when administered at the 10× concentration (Fig. S1 and S2, respectively). These data suggest that TDF alone reduces viability and proliferation of mouse NPCs.

### TDF Induces Apoptosis in Mouse NPCs.

**Fig. 6:**
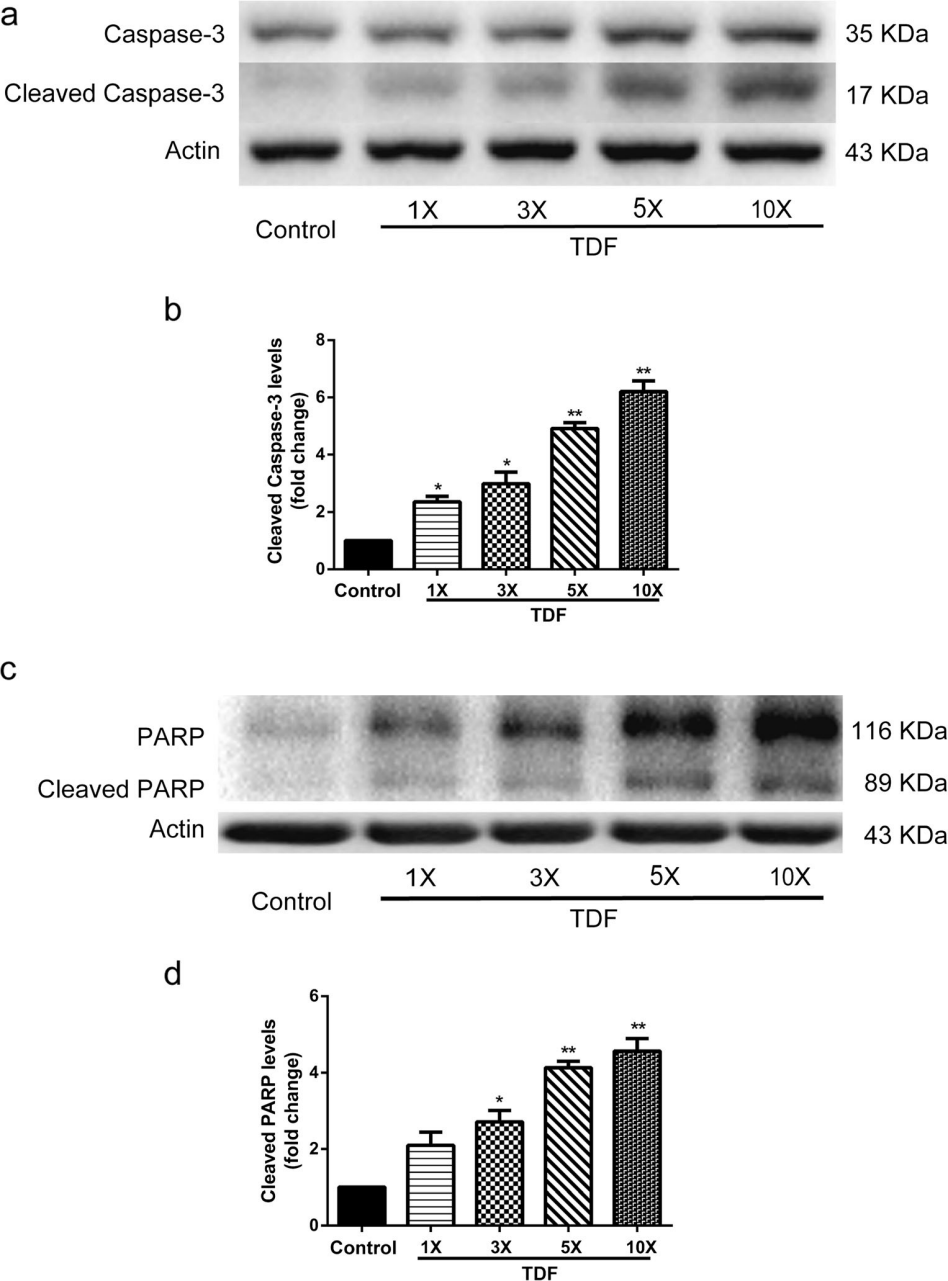
TDF induces apoptosis in mouse NPCs. Mouse NPCs were treated with DMSO or doses of TDF for 8 h. **a** Cleaved Caspase-3 levels were determined by Western blotting. **b** Levels of cleaved Caspase-3 were normalized to the levels of Actin and shown as fold changes relative to the control. **c** Cleaved PARP levels were determined by Western blotting. **d** Levels of cleaved PARP were normalized to the levels of Actin and shown as fold changes relative to the control. * denotes *p* < 0.05, ** denotes *p* < 0.01

We then determined if TDF induces NPC apoptosis by examining the levels of cleaved Caspase-3 and cleaved PARP in cultured mouse NPCs after TDF treatment using Western blotting. We treated mouse NPCs with TDF at multiple concentrations (1×, 3×, 5×, and 10×) for 8 h. Significant increments in levels of cleaved Caspase-3 and cleaved PARP were observed in essentially all TDF treated groups, with the only exception of cleaved PARP in 1× TDF treated group (Fig. 6), indicating that TDF alone also induces apoptosis in mouse NPCs.

### TDF Affects Mouse NPCs in a Dose- and Time-Dependent Manner.

**Fig. 7:**
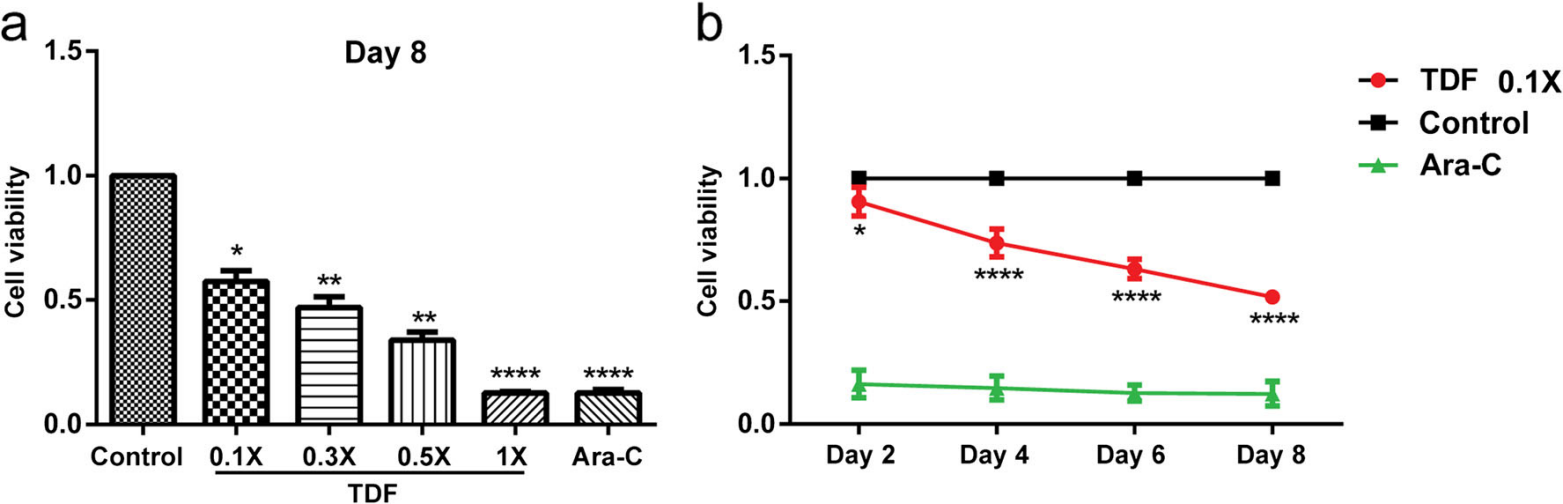
TDF affects mouse NPCs in a dose- and time-dependent manner. Viability of cultured mouse NPCs was determined using the CCK-8. **a** Mouse NPCs were treated with increasing concentrations of TDF (0.1×, 0.3×, 0.5×, and 1×), and cell viability was measured on day 8. **b** Mouse NPCs were treated with 0.1× TDF and cell viability was determined on day 2, 4, 6, and 8. Control groups were treated with DMSO. Ara-C (7 μg/ml) served as a positive control. * denotes *p* < 0.1, ** denotes *p* < 0.01, **** denotes *p* < 0.0001 compared with control

To test if TDF also affects the viability of mouse NPCs in a dose- and time-dependent manner, we treated cultured mouse NPCs with a series of concentrations of TDF (0.1×, 0.3×, 0.5×, and 1×), and calculated cell viability on day 2, 4, 6, and 8 using CCK-8. Like TDF/FTC/RAL combined medication, we found that TDF treatment reduced NPC viability in a dose- and time-dependent manner (Fig. 7).

Taken together, our data show that TDF, among the three drugs used in the combined medication, accounts for most of the effects on NPCs.

## Discussion

In this study, we examined the effects of TDF/FTC/RAL combined medication, a commonly used anti-HIV-1 regimen, on NSCs and progenitors both in vivo and in vitro. Our results show that (1) TDF/FTC/RAL treatment affects NSC homeostasis and progenitor proliferation in the mouse DG; (2) exposure to TDF/FTC/RAL inhibits proliferation and induces apoptosis of cultured mouse NPCs; and (3) TDF, among the three drugs used in this antiretroviral regimen, accounts for most of the effects of combined medication on NPCs.

Why has HAND become increasingly prevalent in AIDS patients who could now live longer because of the effective combination antiretroviral therapy? Our results offer an explanation: Neurogenesis occurs throughout life in the normal adult mammalian brain. In response to brain injuries, NSCs and progenitors have potentials to either replace lost neurons or promote neuronal repair (Aboody et al. 2000; Imitola et al. 2004; Imitola et al. 2003; Park et al. 2002a; Park et al. 2002b; Saha et al. 2013; Snyder et al. 1997). By reducing proliferation and inducing apoptosis of NSCs and progenitors, thereby affecting neurogenesis, antiretroviral treatment exacerbates the brain injuries and cognitive impairment associated with HAND. Consistent with this idea, it has been reported that antiretroviral drugs with good CNS penetration are associated with poor neurocognitive performance of advanced AIDS patients (Marra et al. 2009). Intuitively, enhanced penetration of antiretroviral compounds into the CNS is desired in order to control HIV-1 replication in this reservoir. However, our results suggest that efforts will need to be made to balance the risk of increasing neurotoxicity when targeting antiretroviral drugs to the CNS in the future.

Despite the extensive research on the toxicity of antiretroviral compounds in a variety of cell types, little attention has been paid to the potential deleterious effects of their administration on the nervous system. A previously study showed that antiretroviral compounds including TDF and FTC cause damages in the nervous system such as beading, simplification of the dendritic processes, and neuronal shrinkage (Robertson et al. 2012). Another study showed that in macaque which received early combination antiretroviral therapy including TDF, expression of synaptophysin is significantly decreased in the hippocampi (Akay et al. 2014). Adding to these findings, our data reveals that TDF inhibits proliferation and induces apoptosis of NPCs. Together, these results suggest that TDF may be replaced by another antiretroviral drug in order to reduce the incidence of HAND among AIDS patients.

On the contrary, our results suggest that RAL might be relatively harmless to NPCs: when administered alone, RAL does not seem to affect the viability and proliferation activity of NPCs. This is consistent with previous findings that suggest a low probability of neurotoxicity, and likely a neuroprotective role of RAL during HIV-1 infection (Tatro et al. 2014). In a previous clinical study, a RAL-based anti-HIV-1 therapy maintains a more favorable safety profile than a efavirenz-based therapy, with fewer patients reported with neuropsychiatric side effects and drug-related adverse events (Rockstroh et al. 2013). It has also been reported that RAL does not affect mitochondrial function or compromise viability of cultured rat neurons (Blas-Garcia et al. 2014).

Given that AIDS patients are treated with antiretroviral drugs for years, and even a low dose of these drugs could cause adverse effects on NPCs when administered for a prolonged period of time, it is important to carefully investigate the neurotoxicity of any anti-HIV-1 drug in order to reduce the prevalence of HAND. These studies will greatly benefit the search for improved strategy of combination antiretroviral therapy that will not only effectively suppress HIV replication, but also leave the nervous system intact.

## Electronic supplementary material

Fig. S1 FTC does not reduce viability and proliferation of mouse NPCs. Mouse NPCs were treated with DMSO (**a**) or doses of FTC (**b-d**). After 48 h, cells were fixed and stained with DAPI (blue), anti-Ki67 (green), and anti-Nestin (red) antibodies. Quantification of total cell number was shown in (**e**). Quantification of the ratio between Ki67-positive and DAPI-positive cells was shown in (**f**). Scale bar: 100 μm (GIF 187 kb)

High resolution image (TIFF 31857 kb)

Fig. S2 RAL does not reduce viability and proliferation of mouse NPCs. Mouse NPCs were treated with DMSO (**a**) or doses of RAL (**b-d**). After 48 h, cells were fixed and stained with DAPI (blue), anti-Ki67 (green), and anti-Nestin (red) antibodies. Quantification of total cell number was shown in (**e**). Quantification of the ratio between Ki67-positive and DAPI-positive cells was shown in (**f**). Scale bar: 100 μm (GIF 187 kb)

High resolution image (TIFF 30177 kb)
