# Supplementary figures and images for: Combined Medication of Antiretroviral Drugs Tenofovir Disoproxil Fumarate, Emtricitabine, and Raltegravir Reduces Neural Progenitor Cell Proliferation In Vivo and In Vitro
Source: J Neuroimmune Pharmacol. 2017 Jul 22;12(4):682–92. doi: 10.1007/s11481-017-9755-4 (PMC5693968; doi:10.1007/s11481-017-9755-4)

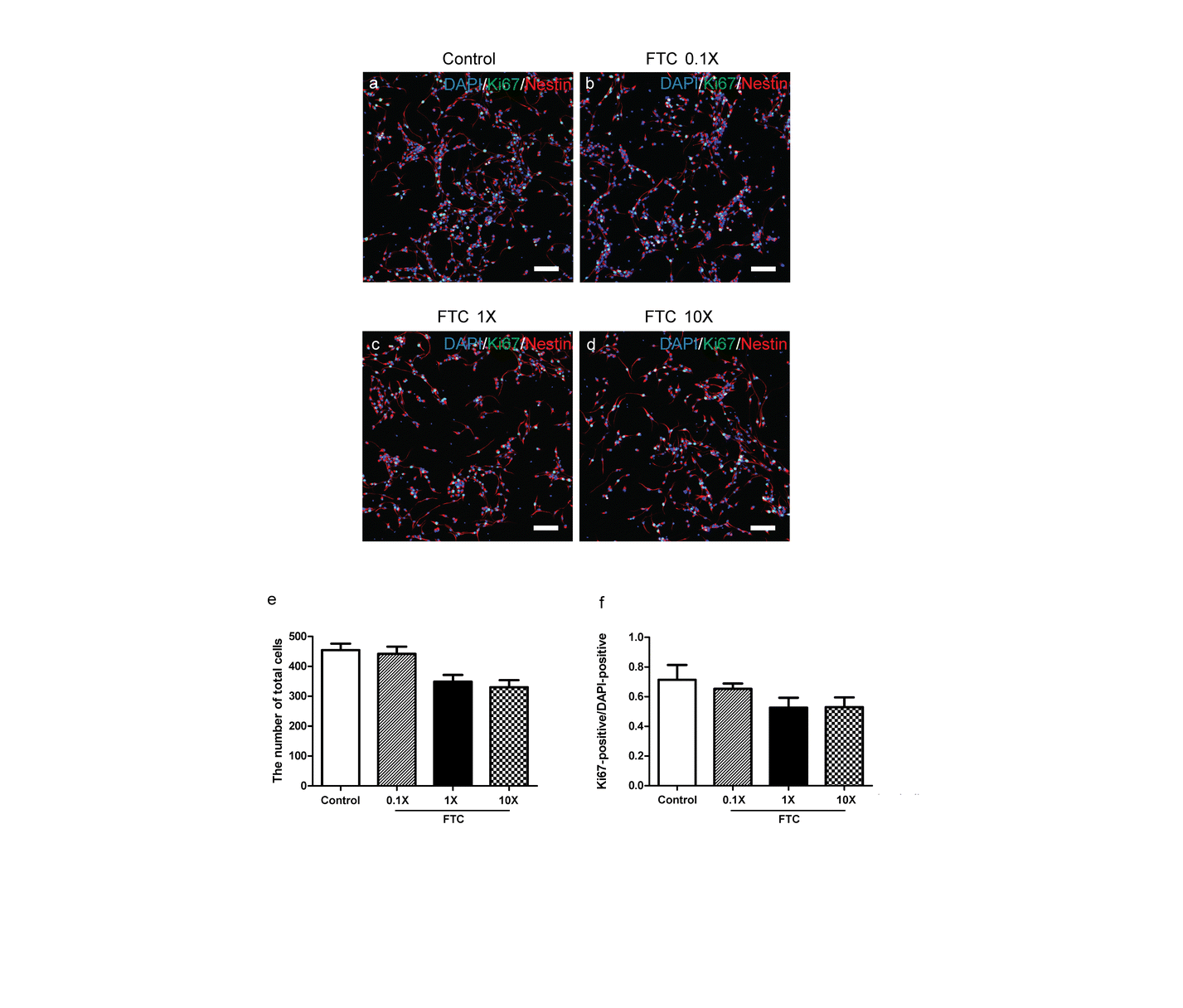

Supplement: Supplementary file 1 — FTC does not reduce viability and proliferation of mouse NPCs. Mouse NPCs were treated with DMSO (a) or doses of FTC (b-d). After 48 h, cells were fixed and stained with DAPI (blue), anti-Ki67 (green), and anti-Nestin (red) antibodies. Quantification of total cell number was shown in (e). Quantification of the ratio between Ki67-positive and DAPI-positive cells was shown in (f). Scale bar: 100 μm (GIF 187 kb) [file 11481_2017_9755_Fig8_ESM.gif]

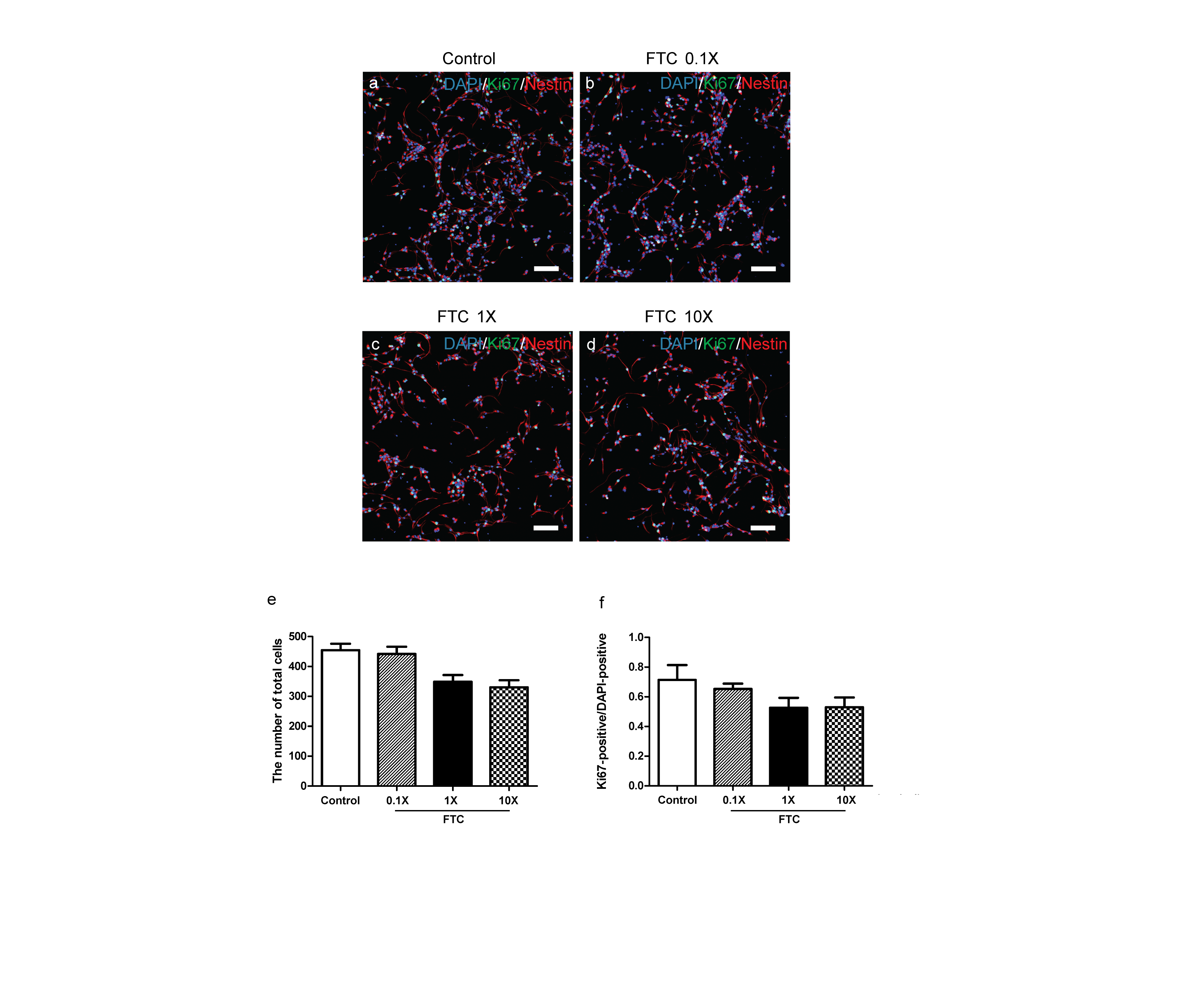

Supplement: Supplementary file 2 — High resolution image (TIFF 31857 kb) [file 11481_2017_9755_MOESM1_ESM.tif]

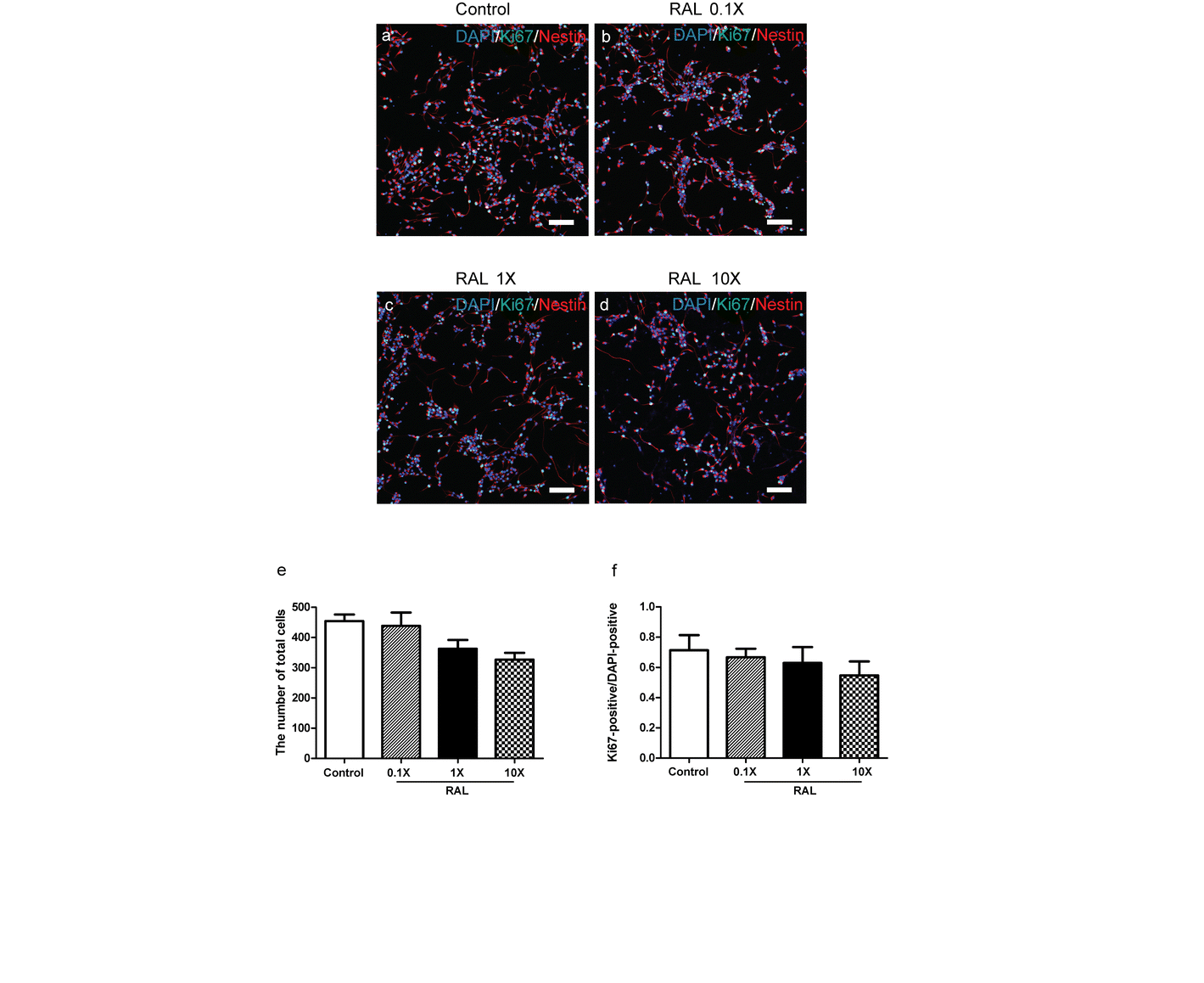

Supplement: Supplementary file 3 — RAL does not reduce viability and proliferation of mouse NPCs. Mouse NPCs were treated with DMSO (a) or doses of RAL (b-d). After 48 h, cells were fixed and stained with DAPI (blue), anti-Ki67 (green), and anti-Nestin (red) antibodies. Quantification of total cell number was shown in (e). Quantification of the ratio between Ki67-positive and DAPI-positive cells was shown in (f). Scale bar: 100 μm (GIF 187 kb) [file 11481_2017_9755_Fig9_ESM.gif]

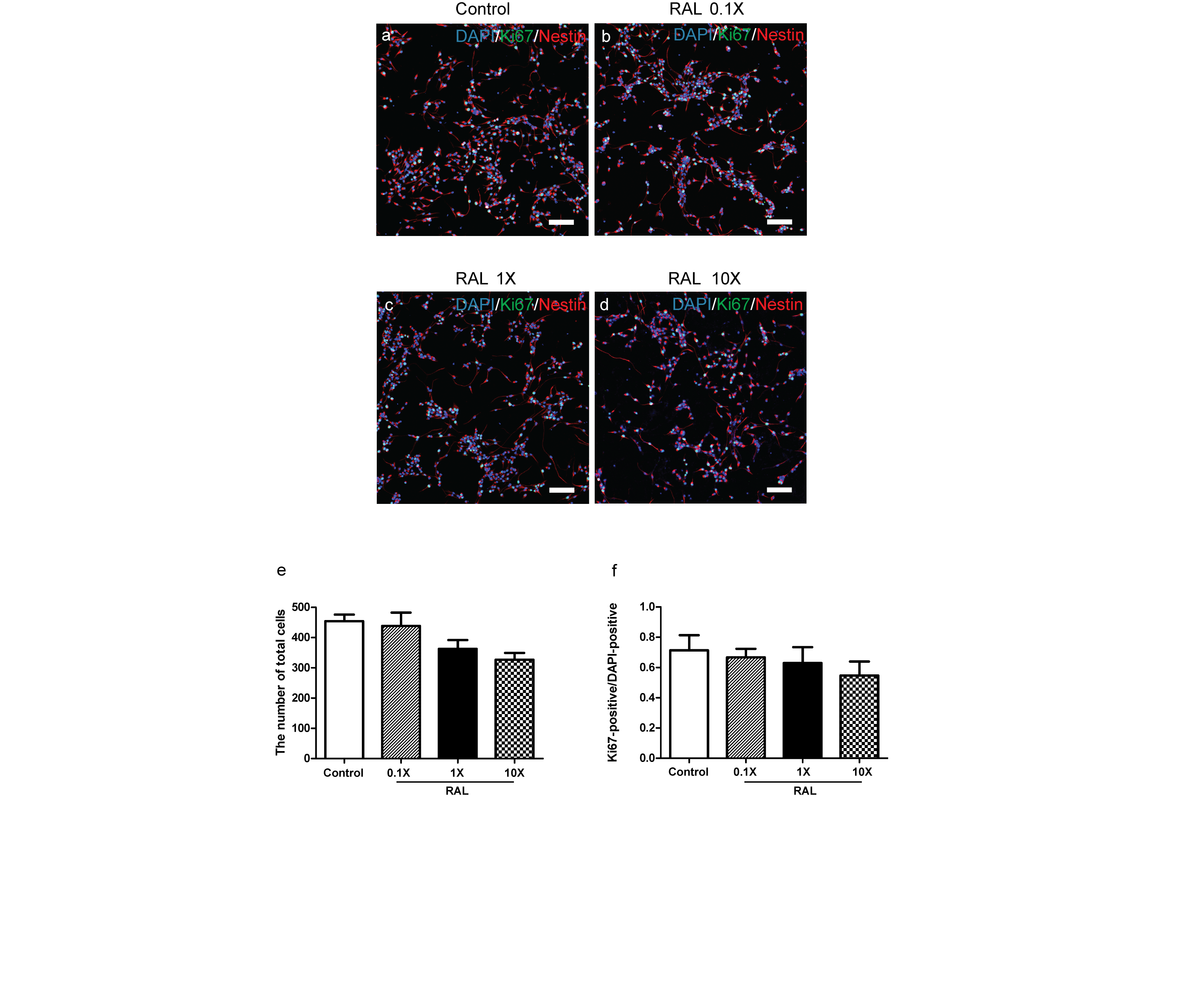

Supplement: Supplementary file 4 — High resolution image (TIFF 30177 kb) [file 11481_2017_9755_MOESM2_ESM.tif]
